# Supplementary figures and images for: Effects of Guangzhou seasonal climate change on the development of Aedes albopictus and its susceptibility to DENV-2
Source: PLoS One. 2022 Apr 1;17(4):e0266128. doi: 10.1371/journal.pone.0266128 (PMC8975156; doi:10.1371/journal.pone.0266128)

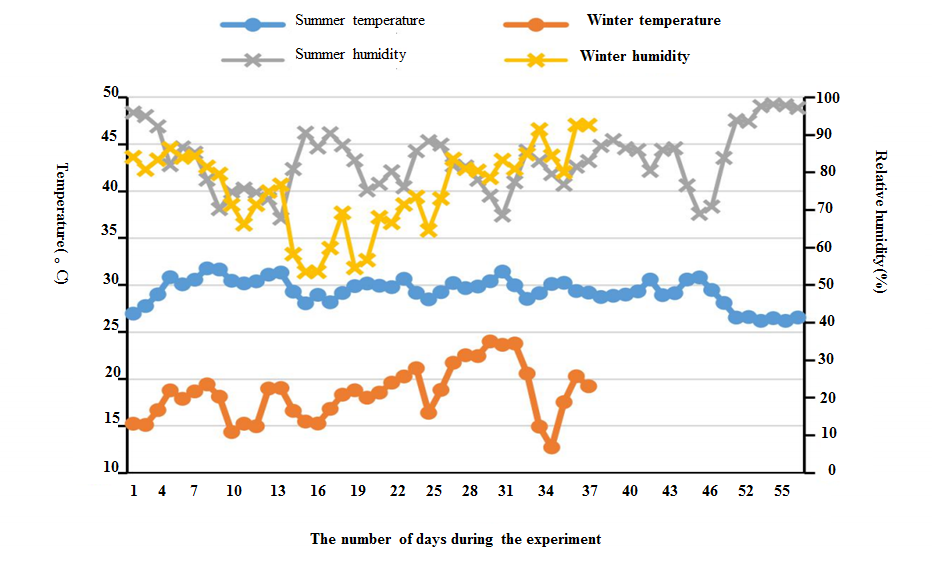

Supplement: S1 Fig — (TIF) [file pone.0266128.s001.tif]

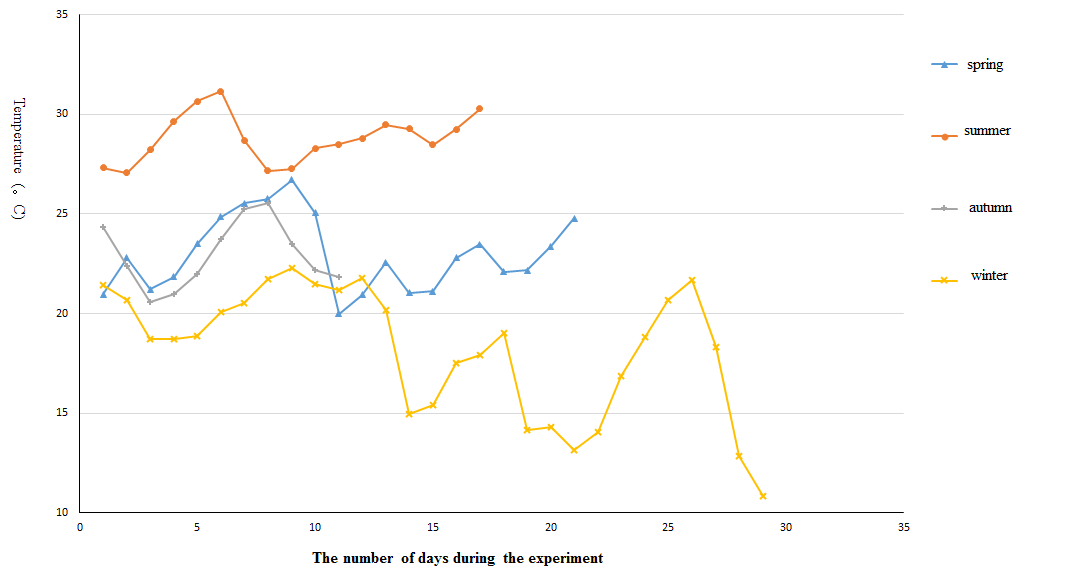

Supplement: S2 Fig — (TIF) [file pone.0266128.s002.tif]

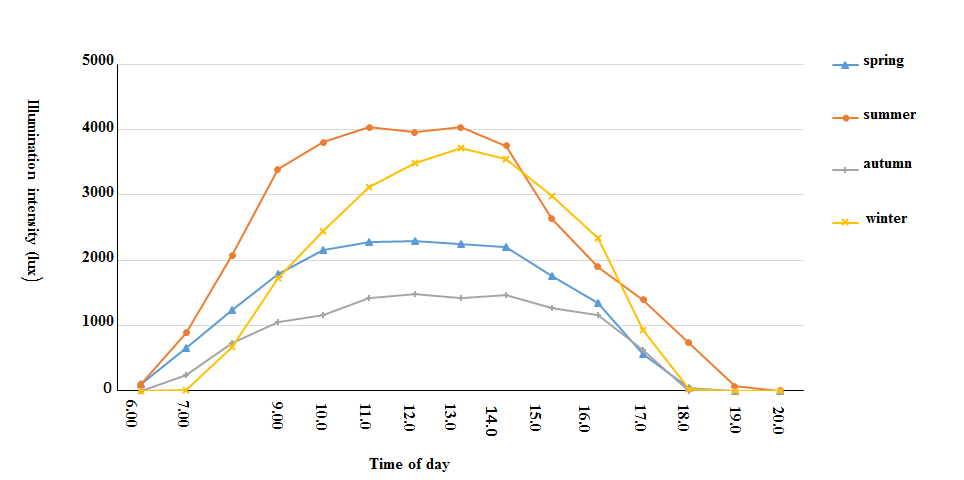

Supplement: S3 Fig — (TIF) [file pone.0266128.s003.tif]

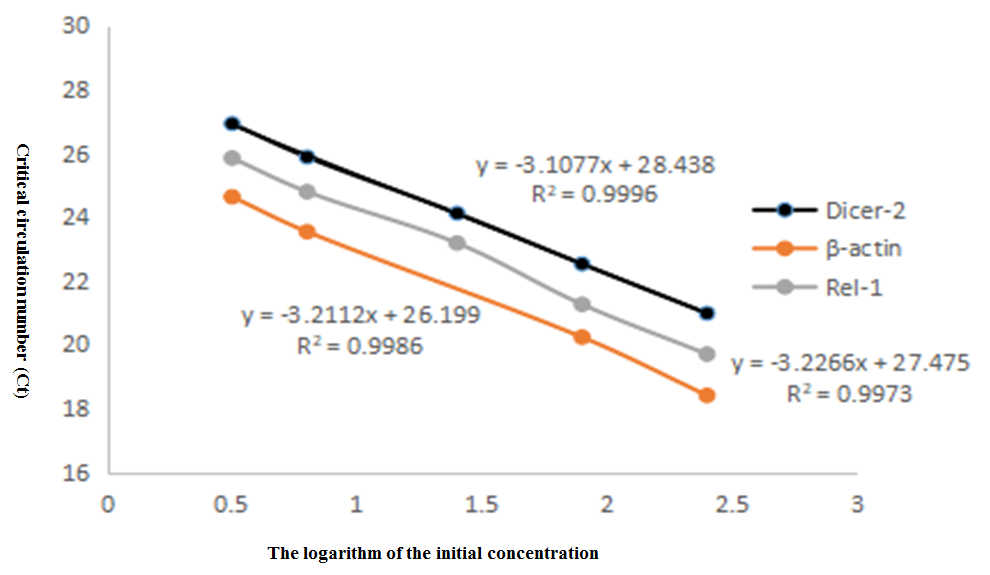

Supplement: S4 Fig — (TIF) [file pone.0266128.s004.tif]

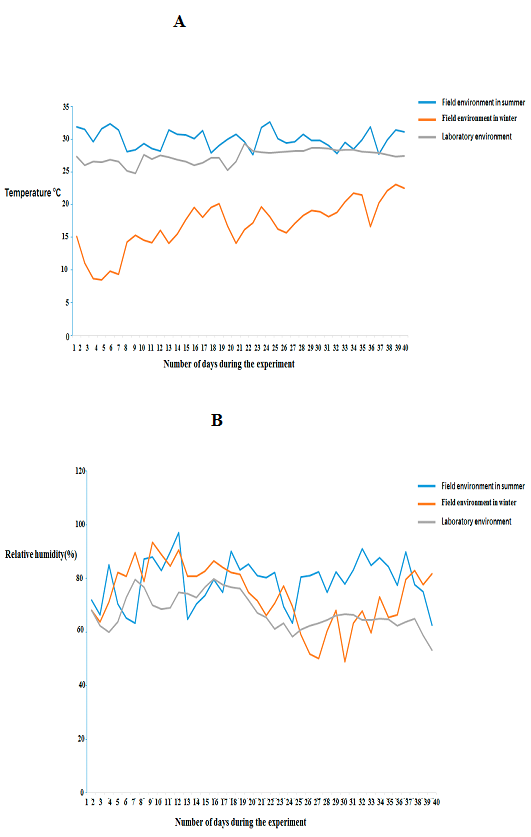

Supplement: S5 Fig — A: Temperature changes during the experiment in different environments. B: Variation of relative humidity in different environments. (TIF) [file pone.0266128.s005.tif]

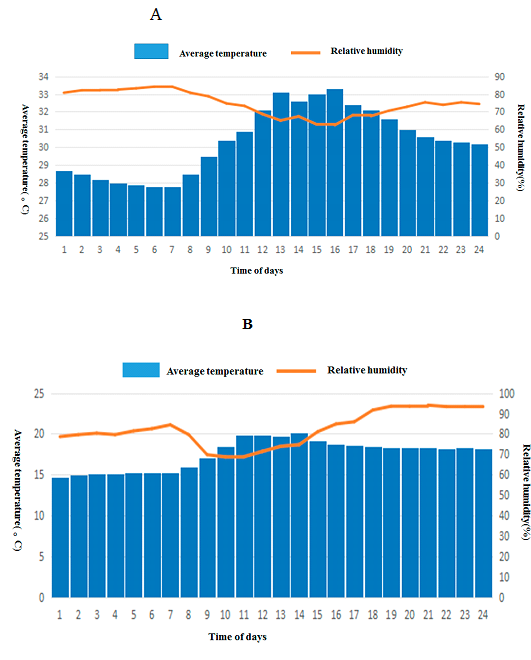

Supplement: S6 Fig — A: Daily average temperature and relative humidity. B: Average temperature and relative humidity per hour. (TIF) [file pone.0266128.s006.tif]
