# Supplementary material for: Effects of Guangzhou seasonal climate change on the development of Aedes albopictus and its susceptibility to DENV-2
Source: PLoS One. 2022 Apr 1;17(4):e0266128. doi: 10.1371/journal.pone.0266128 (PMC8975156; doi:10.1371/journal.pone.0266128)
Supplement: S1 Table — (DOCX) [file pone.0266128.s007.docx]

S1 Table. Hatching results of *Ae. albopictus* eggs under different environmental conditions

| Experimental group | N  (n x replicates) | Hatch rate  (%) | Hatch time (days) |
| --- | --- | --- | --- |
| Laboratory | 600 (200 x 3) | 84.5 ± 3.1 | 1.75 ± 0.18 |
| Summer experiment | 600 (200 x 3) | 73.7 ± 2.8 | 2.41 ± 0.08 |
| Winter experiment | 600 (200 x 3) | 59.0 ± 3.9 | 6.17 ± 0.70 |
