# Supplementary material for: Effects of Guangzhou seasonal climate change on the development of Aedes albopictus and its susceptibility to DENV-2
Source: PLoS One. 2022 Apr 1;17(4):e0266128. doi: 10.1371/journal.pone.0266128 (PMC8975156; doi:10.1371/journal.pone.0266128)
Supplement: S2 Table — (DOCX) [file pone.0266128.s008.docx]

S2 Table. Pupation rate and pupation time of *Ae. albopictus* larvae under different environmental conditions

| Experimental group | N  (larvae, 3 replicates) | Pupation rate  (%) | Pupation time (days) |
| --- | --- | --- | --- |
| Laboratory | 506 | 89.1 ± 0.7 | 8.2 ± 0.2 |
| Summer experiment | 442 | 73.7 ± 2.8 | 7.2 ± 0.02 |
| Winter experiment | 356 | 59.0 ± 3.9 | 20.5 ± 0.7 |
