# Supplementary material for: Effects of Guangzhou seasonal climate change on the development of Aedes albopictus and its susceptibility to DENV-2
Source: PLoS One. 2022 Apr 1;17(4):e0266128. doi: 10.1371/journal.pone.0266128 (PMC8975156; doi:10.1371/journal.pone.0266128)
Supplement: S3 Table — (DOCX) [file pone.0266128.s009.docx]

S3 Table. Eclosion results of *Ae. albopictus* pupae under different environmental conditions

| Experimental group | N  (pupae, 3 replicates) | Ecolosion rate  (%) | Female proportion (%) |
| --- | --- | --- | --- |
| Laboratory | 596 | 93.4 | 50.7 ± 7.1 |
| Summer experiment | 543 | 98.9 | 51.4 ± 6.5 |
| Winter experiment | 465 | 94.2 | 47.9 ± 2.2 |
