# Supplementary material for: Effects of Guangzhou seasonal climate change on the development of Aedes albopictus and its susceptibility to DENV-2
Source: PLoS One. 2022 Apr 1;17(4):e0266128. doi: 10.1371/journal.pone.0266128 (PMC8975156; doi:10.1371/journal.pone.0266128)
Supplement: S4 Table — (DOCX) [file pone.0266128.s010.docx]

S4 Table. Oviposition of adult *Ae. albopictus* under different environmental conditions

| Experimental group | N  (adults, F/M) | Eggs/female | Oviposition time (average, range, days) |
| --- | --- | --- | --- |
| Laboratory | 90/90 | 75.9 ± 8.6 | 7.1 ± 5.4 (3-29) |
| Summer experiment | 90/90 | 137.4 ± 29.0 | 20.4 ± 2.2 (5-54) |
| Winter experiment | 60/60 | 4.7 ± 4.2 | 18.2 ± 1.6 (13-25) |

** Range: the earliest and last day of oviposition observed
