# Supplementary material for: Effects of Guangzhou seasonal climate change on the development of Aedes albopictus and its susceptibility to DENV-2
Source: PLoS One. 2022 Apr 1;17(4):e0266128. doi: 10.1371/journal.pone.0266128 (PMC8975156; doi:10.1371/journal.pone.0266128)
Supplement: S6 Table — (DOCX) [file pone.0266128.s012.docx]

S6 Table The expression of Dicer-2 gene and Rel-1 gene in the midguts of *Aedes albopictus* in 7 and 14 days-post-infection (dpi) in summer and winter experiments

| Experimental group Dicer-2 Rel-1 |
| --- |
| The 7th day of Infection group in  simulated summer field environment 1.1 1.1  The 14th day of Infection group in  simulated summer field environment 1.7 1.5  The 7th day of Infection group in  simulated winter field environment 4.1* 1.4  The 14th day of Infection group in  simulated winter field environment 1.9 1.6 |

* Representing significantly different from null (unit) at level of 0.05
