# Supplementary material for: Effects of Guangzhou seasonal climate change on the development of Aedes albopictus and its susceptibility to DENV-2
Source: PLoS One. 2022 Apr 1;17(4):e0266128. doi: 10.1371/journal.pone.0266128 (PMC8975156; doi:10.1371/journal.pone.0266128)
Supplement: Attachment — Submitted filename: Responses to reviewers2022.2.5.docx [file pone.0266128.s014.docx]

Department of Pathogen Biology

School of Public Health

Southern Medical University

1838 Guangzhou North Av.

Guangzhou 510515, China

Tel: +86-20-6164-8651

E-mail: zhengxueli2001@126.com

On February 5, 2022

Dear PLOSE ONE Editor:

Thank you very much for your email dated on 2020/14/1 with comments attached for our manuscript ((PONE-D-21-26984). We have now made a point-to-point response to all reviewers and Editor (Rafael Maciel-de-Freitas Academic Editor) comments. All page and line numbers described below refer to the revised version.

All contributing authors have reviewed and approved this revised version. The material has not been and will not be offered elsewhere for publication. Please let me know if further editorial or clarifications need to be made. The authors declare that they have no competing interests.

Thank you very much for your assistance with this paper.

Yours sincerely,

Xueli Zheng, Ph.D.

Professor

**Responses to reviewers #1:**

Reviewer #1: The article has potential. But the introduction is extensive and misses the main subject. The hypothesis and the objectives are not clear. The Methods and Results of the statistical tests are not clear. The statistical results presented in the table and figures are not making sense, as well as the groups compared. The four stations appear in the group "environment" and "laboratory", but this description of the result is incompatible with the methodology.... The article has basic problems for answer the objective, such as the description of temperatures and humidity in each group compared.

**Response:** We sincerely thank the reviewer’s positive comment. We agree with you and have made relevant modifications.

Reviewer #2: Suggestions to improve clarity.

**Response:** We sincerely thank the reviewer’s positive comment. We agree with you and have made relevant modifications.

(1) Methods.
- Line 193: “Larvae were brought back to semi-field setting and reared in microcosms where life table experiments were conducted”. Please, provide a more detailed description of the “microcosm” used to rear the mosquitoes.

**Response:** We have revised it. The field environment was set in Southern Medical University campus of Baiyun District in Guangzhou. The garden was closed and managed by special personnel to avoid interference by irrelevant human factors. The environment for raising *Ae. albopictus* in the field comprised mosquito nets placed in the pavilions with sunshade roofs and ventilation around. The plants in the garden are luxuriant, which was conducive to the breeding of *Ae. albopictus*. The laboratory environment was set up in the standard insect breeding room. All larvae and emerged adults were maintained in microcosms covered with mesh to prevent the adult mosquitoes from escaping. Mosquito netting was placed over all microcosms as further protection. The laboratory conditions were temperature: 27 ± 1 °C, humidity: 70–80%, light cycle, day:night = 14h:10 h.

In the field environment group(Southern Medical University campus of Baiyun District in Guangzhou), the experiments were conducted inside a shed with open, screened doors and windows, under natural conditions[13] , an environment similar to that described in previous studies [13]. All larvae and emerged adults were maintained in microcosms covered with mesh to prevent the adult mosquitoes from escaping. Mosquito netting was placed over all microcosms as further protection.(

(2) Line 196: “F3 eggs were used for first round of life-table experiments for three reasons, to allow for get enough eggs within one day, to allow for field mosquitoes to adapt the new environment and mouse blood.”. Please, restructure this sentence to improve clarity.
**Response:** We've rewritten this paragraph.

(3) Line 203: Is it possible to use another title for this section? It is not clear to me what the authors mean by “Semi-Life scale experimental methods”.

**Response:** We've rewritten this paragraph.

(4) Line 250. It is not clear to me the section “Expanded culture of experimental mosquitoes”, including the title. Particularly the last sentence “In July 2019, eggs of Ae. albopictus from Guangzhou were released in both the field and laboratory environments.”.
**Response:** We've rewritten this paragraph.

(5) - Line 275. “the mosquito was placed a glass dish on ice”. Please, correct the sentence.
We have revised it. the mosquito was Anesthetized using by putting it on ice.

(6) - 288 to 292. Overall I think the description of study design is not straightforward. The section described from line 288 to 292 is an example.
**Response: Thank your suggestion,** We've rewritten this sentences.

(7) - Line 317. “cDNA was synthesized using a DENV-2-specific primer (5'-TGGTCTTTCCCAGCGTCAAT-3')”. Is this correct? Only one primer was used? Please, clarity.

cDNA was synthesized using a DENV-2-specific primer (5'-TGGTCTTTCCCAGCGTCAAT-3'), and the recommendations of the GoScript™ Reverse Transcription System (Promega, Madison, WI, USA) were followed.

Polymerase chain reaction (PCR) was used to detect DENV-2 in tissues. A pair of primers was synthesized as described previously (forward primer: 5'-TCAATATGCTGAAACGCGC GAGAAACCG-3'; reverse primer: 5’-TTGCACCAACAGTCAATGTCTTCAGGTTC-3') [27].

**Response:** cDNA was synthesized using a DENV-2-specific primer (5'-TGGTCTTTCCCAGCGTCAAT-3'), and the recommendations of the GoScript™ Reverse Transcription System (Promega, Madison, WI, USA) were followed. We've check this sentences.

(8) Results:
- Line 461. Figure 2. Study design was not clear to me, especially what “control” means. Authors could use figure legends to clarify.

**Response: Thank your suggestion,** We've rewritten this paragraph.

The experimental group was divided into the laboratory environment group and the field environment group. The field environment group was divided into four groups, spring field environment group(spring), summer field environment group (summer), autumn field environment group(autumn), winter field environment group (winter). The laboratory environment group was also divided into four groups, spring group, summer group, autumn group, winter group.

In the field environment, the pupation rates of *Ae. albopictus* larvae of Guangzhou strain and laboratory strain were the lowest in spring, at 85.0% and 83.3% respectively; however, Guangzhou strain, the pupation rates of *Ae. albopictus* larvae in summer, autumn, and winter were more than 95.0%. In the field, the emergence rates of the Guangzhou strain and laboratory strain were more than 80.0% all year round. The emergence rates of the two strains reached the highest in summer, at 93.3% and 95.0% respectively, followed by autumn (Figure.3A, in the field environment).

In the laboratory environment, the pupation rates of the Guangzhou strain and laboratory strain were the lowest in spring, at 80.0%, and the pupation rates of the Guangzhou strain in summer, autumn, and winter were more than 90.0%. The pupation rates of the laboratory strains in summer and autumn were more than 95.0%, while that in winter was 86.7%. In the laboratory environment, the emergence rates of the Guangzhou strain in summer, autumn, and winter were more than 90.0%, and were the lowest in spring at 78.3%. The emergence rates of the laboratory strain in summer and autumn were more than 95.0%, and were lower in spring and winter, at 72.5% and 71.7%, respectively (figure.3B, in the laboratory environment).

(9)Line 525. Figure 5. Please, add number of mosquitos analyzed in figure legends, so readers can at least average egg/mosquito without having to search for this information in the text. Please, define in figure legends what authors mean by “control”.

**Response: Thank your suggestion, We ‘ve revised it.**

(10)
- Line 655. I could not find figure 9C and 9D.

**Thank your suggestion, We ‘ve revised it.**
 (11) Discussion:

- Line 858. Please, restructure sentence to improve clarity.

**Thank your suggestion, We ‘ve revised it.**
